# Risk factors for recurrence and surgical site infection after abdominal wall hernia repair: a systematic review and meta analysis

**DOI:** 10.3389/fsurg.2026.1835070

**Published:** 2026-06-03

**Authors:** Ping Wei, Xin Zhao, Guangjian Tian

**Affiliations:** General Surgery Department, Beijing Luhe Hospital, Capital Medical University, Beijing City, China

**Keywords:** abdominal wall hernia, meta-analysis, recurrence, risk factors, surgical site infection

## Abstract

**Background:**

Surgical site infection (SSI) and hernia recurrence are among the most common and impactful complications following abdominal wall hernia repair. Identifying patient-related risk factors is essential for improving outcomes and guiding perioperative management.

**Methods:**

A systematic review and meta-analysis were conducted in accordance with PRISMA 2020 and AMSTAR 2 guidelines. PubMed, Embase, Scopus, CNKI,Wanfang, and VIP were searched up to September 20,2025. Eligible studies included randomized controlled trials, cohort, and case-control studies that reported associations between patient-related variables and SSI or recurrence after hernia repair. Pooled odds ratios (ORs) with 95% confidence intervals (CIs) were calculated using random-effects models. Beyond I^2^, the 95% prediction interval (PI) was utilized as the primary metric to evaluate the absolute dispersion of true effects. Risk of bias was assessed using the Newcastle–Ottawa Scale (NOS).

**Results:**

A total of 11 studies involving 97,428 patients were included. No significant associations were observed for female sex (OR: 1.05, 95% CI: 0.77–1.42, *P* = 0.77; 95% PI: 0.48–2.29), obesity (OR: 1.15, 95% CI: 0.78–1.68, *P* = 0.48; 95% PI: 0.37–3.55), COPD (OR: 0.95, 95% CI: 0.47–1.92, *P* = 0.88; 95% PI: 0.14–6.25), or immunosuppressive therapy (OR 0.90, 95% CI: 0.38–2.14, *P* = 0.81). Although diabetes mellitus reached nominal statistical significance (OR: 1.46, 95% CI: 1.01–2.11, *P* = 0.04), its wide 95% PI (0.55–3.90) suggested considerable inconsistency across cohorts. Similarly, higher ASA classification (>3 vs. <2; OR: 1.63, 95% CI: 0.96–2.78, *P* = 0.07; 95% PI: 0.47–5.69) was not a consistent predictor. Data on recurrence were fragmented and unsuitable for robust pooled analysis, although individual studies suggested that risk factors for recurrence may differ from those for SSI.

**Conclusions:**

Contrary to common clinical assumptions, traditional host-related risk factors such as gender, obesity, and diabetes do not consistently predict adverse outcomes after abdominal wall hernia repair when clinical heterogeneity is rigorously accounted for. The wide 95% PIs observed for most factors underscore substantial clinical variance across surgical settings. These findings highlight the limitations of relying on isolated comorbidities for risk assessment and advocate for the development of integrated, individualized risk prediction models.

**Systematic Review Registration:**

https://www.crd.york.ac.uk/prospero/display_record.php?ID=CRD420261278753, identifier CRD420261278753.

## 1 Introduction

Abdominal wall hernia is one of the most common conditions in general surgery, with millions of hernia repair procedures performed worldwide each year (1, 2). With the rising burden of population aging and obesity-related diseases, the number of operations continues to increase (3). Despite advances in surgical techniques (such as laparoscopy and robotic-assisted surgery) and mesh materials (lightweight, composite, and biologic meshes), surgical site recurrence and surgical site infection (SSI) remain the major adverse outcomes (4, 5). These complications substantially increase the risks of reoperation and rehospitalization, prolong recovery time, raise healthcare costs, and exert long-lasting negative effects on patients' quality of life (6). The incidence of recurrence and SSI is particularly high in incisional hernias and complex abdominal wall reconstructions, where the clinical and economic burden is especially pronounced.

The occurrence of recurrence and SSI is based on multifactorial and multilayered pathophysiological mechanisms, involving host factors, defect and anatomical characteristics, surgical strategies and implanted materials, as well as perioperative management (7, 8). At the host level, female (possibly related to collagen composition and hormonal status), obesity (increased intra-abdominal pressure, elevated wound tension, and tissue hypoxia), diabetes mellitus (accumulation of advanced glycation end-products, microvascular disease, impaired phagocytic function), immunosuppression (deficiency of innate and adaptive immunity), chronic obstructive pulmonary disease (COPD, in which chronic coughing repeatedly elevates intra-abdominal pressure), and higher American Society of Anesthesiologists (ASA) classification (>3, indicating reduced physiological reserve) are all associated with adverse outcomes (9, 10). Surgical factors include incision and approach (open vs. laparoscopic/robotic), defect size and margin quality, mesh type (synthetic vs. biologic, lightweight vs. heavyweight, absorbable vs. permanent), mesh plane and coverage (onlay, sublay/retro-rectus, IPOM), defect closure vs. bridging, and fixation methods (11, 12). Perioperative factors such as prolonged operative time, wound contamination level (CDC class I–IV), emergency surgery, drain placement, adequacy and timing of antibiotic prophylaxis, intraoperative hypothermia, and poor perioperative glycemic control also contribute to the risk of SSI and recurrence (13–15).

It is important to emphasize that SSI and recurrence may interact with each other. SSI can impair tissue integration, induce chronic inflammation, or necessitate mesh removal, thereby markedly increasing the likelihood of recurrence (16). Conversely, recurrence or abnormal tensile stress may impair skin and soft tissue perfusion, further predisposing to infection and forming a vicious cycle (17). Moreover, different hernia types carry distinct baseline risks and complication patterns: inguinal hernia is more often associated with neuralgia and early infection, whereas incisional and umbilical hernias are more susceptible to mesh-related complications, SSI, and long-term recurrence (18, 19). Therefore, controlling clinical and methodological heterogeneity is essential when synthesizing evidence across various hernia types and surgical techniques (19). Although multiple studies have investigated potential risk factors, the evidence remains inconsistent and methodologically limited. First, outcome definitions vary recurrence may be assessed by physical examination, imaging (ultrasound or CT), or reoperation records, with inconsistent follow-up durations; SSI definitions and classifications (CDC superficial, deep, or organ/space) are applied inconsistently, with variable observation windows, limiting comparability. Second, confounding and bias are common: observational studies are prone to indication bias, selection bias, and information bias, with inconsistent adjustment for key covariates. Moreover, surgical strategy, mesh use, and perioperative optimization are often implemented as “treatment bundles,” making it difficult to isolate the independent effects of specific factors. Third, prior quantitative syntheses have often focused on a single hernia type or a single risk factor, lacking comprehensive evaluation of both recurrence and SSI within a unified analytical framework. Furthermore, systematic subgroup analyses (e.g., open vs. laparoscopic/robotic approach, mesh plane, contamination class, elective vs. emergency surgery) and sensitivity analyses remain insufficient.

Against this background, the present study aims to conduct a systematic review and meta-analysis, in accordance with Preferred Reporting Items for Systematic Reviews and Meta-Analyses (PRISMA) 2020 guidelines, to comprehensively synthesize the available evidence and quantitatively assess the main risk factors for recurrence and SSI after abdominal wall hernia repair. Particular attention is given to host-related variables such as female, obesity (BMI), diabetes mellitus, use of immunosuppressive agents, COPD, and ASA classification (>3 vs. <2), while surgical strategies, mesh characteristics, and perioperative management factors are incorporated when available. This study aims to identify potentially modifiable risk factors associated with surgical site infection (SSI) and hernia recurrence, providing evidence to support preoperative risk stratification and perioperative optimization strategies.

## 2 Methods

### 2.1 Protocol

This systematic review and meta-analysis were conducted in accordance with the PRISMA 2020 guidelines (20). Furthermore, to ensure methodological rigor, the study design and reporting strictly adhered to the AMSTAR 2 (Assessing the Methodological Quality of Systematic Reviews) guidelines (21).

### 2.2 Eligibility criteria

Studies were included based on predefined criteria guided by the PICO (Population, Intervention, Comparator, Outcome) framework. The study population included patients undergoing abdominal wall hernia repair, which defined broadly to include ventral (umbilical, epigastric), incisional, and groin (inguinal) hernias. The exposure or intervention of interest was the presence of potential risk factors (e.g., patient comorbidities, surgical technique, perioperative management) associated with postoperative recurrence or SSI. Comparator groups consisted of individuals without specified exposures or with varying levels of exposure (e.g., low vs. high BMI, laparoscopic vs. open approach). The primary outcomes were hernia recurrence and SSI incidence. Eligible study designs included randomized controlled trials (RCTs), cohort studies (prospective or retrospective), and case-control studies. No restrictions were applied regarding publication status, language, or geographical location.

### 2.3 Search strategy

A comprehensive and systematic literature search was conducted across multiple electronic databases, including PubMed, Embase, Web of Science, Scopus, China National Knowledge Infrastructure (CNKI), and Wanfang Data, from database inception to September 20, 2025. The search strategy combined relevant Medical Subject Headings (MeSH) and keywords such as “hernia repair”, “abdominal wall hernia”, “recurrence”, “surgical site infection”, and “risk factors”, using Boolean operators (AND, OR) to optimize sensitivity and specificity. The detailed search strategies for each database are presented in Appendix A. Additional manual screening of reference lists from relevant studies was performed to identify further eligible studies.

### 2.4 Study selection and data extraction

All retrieved articles were imported into EndNote reference management software to remove duplicates. Subsequently, two reviewers independently screened the titles and abstracts to identify potentially eligible studies. Full texts of the selected studies were then reviewed in accordance with the predefined inclusion criteria. Any discrepancies were resolved through discussion, and a third reviewer was consulted when consensus could not be reached. Data extraction was also conducted independently by two reviewers using a standardized data collection form. The extracted information included the first author, year of publication, country, sample size, basic patient characteristics (such as age and gender), and relevant clinical variables. The primary risk factors of interest included female, body mass index (BMI, particularly obesity), diabetes mellitus (DM), use of immunosuppressive agents, COPD, and American Society of classificationASA (>3 vs. <2). For studies that reported effect estimates, the odds ratios (ORs) along with their 95% confidence intervals (CIs) were extracted.

### 2.5 Risk of bias assessment

The risk of bias in the included studies was independently assessed by two reviewers. For randomized controlled trials, the Cochrane Risk of Bias tool (RoB 2.0) (22) was used to evaluate domains such as random sequence generation, allocation concealment, blinding, and completeness of outcome data. For observational studies, the Newcastle-Ottawa Scale (NOS) (23) was applied, assessing selection, comparability, and outcome/exposure domains. Any disagreements were resolved through discussion or consultation with a third reviewer to ensure consistency and objectivity.

### 2.6 Statistical analysis

Meta-analyses were performed exclusively using random-effects models to account for inherent between-study variations in clinical studies involving human subjects, as the assumption of a single true effect size in fixed-effect models was deemed inappropriate. For all binary outcomes, the odds ratios (ORs) and their corresponding 95% confidence intervals (CIs) were log-transformed prior to pooling to ensure symmetry, and the results were exponentiated for clinical interpretation. Heterogeneity was assessed using the I^2^ statistic and Cochran's *Q*-test. Following Borenstein's methodology, the 95% Prediction Interval (PI) was calculated and utilized as the primary metric for evaluating heterogeneity. The PI describes the absolute dispersion of true effects and indicates the range within which the effect size of a future study is expected to fall. Sensitivity analyses were conducted by the leave-one-out method to assess the stability of the pooled estimates. Publication bias was examined through Egger's regression test. All statistical analyses were conducted using R4.5.0.

## 3 Results

### 3.1 Study selection

**Figure 1:**
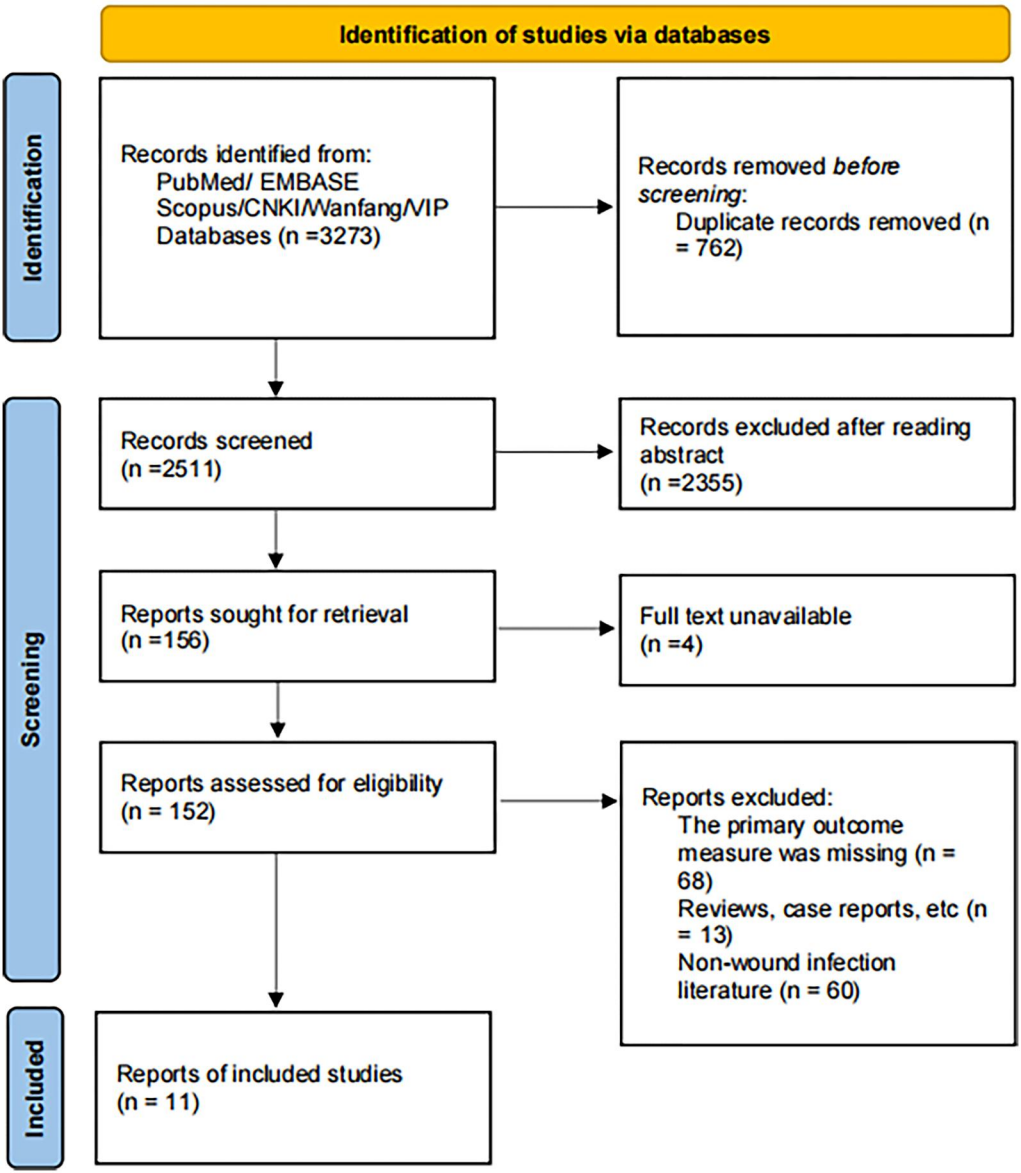
PRISMA 2020 flow diagram of the study selection process.

A total of 3,273 records were identified through database searches (PubMed, EMBASE, Scopus, CNKI, Wanfang, and VIP). After removal of 762 duplicates, 2,511 records remained for title and abstract screening. Of these, 2,355 records were excluded for irrelevance, leaving 156 full-text articles to be retrieved. Four articles were unavailable in full text, and 152 articles were assessed for eligibility. Following detailed evaluation, 141 articles were excluded due to missing primary outcomes (*n* = 68), review articles or case reports (*n* = 13), and non–wound infection literature (*n* = 60). Ultimately, 11 studies met the inclusion criteria and were included in the qualitative synthesis. The study selection process is summarized in the PRISMA 2020 flow diagram (Figure 1).

### 3.2 Study characteristics

Eleven studies (2010–2025) (8–10, 12, 14, 15, 24–28) involving 97,428 patients were included. Cohort sizes ranged from fewer than 200 to over 30,000. Most patients were middle-aged or older, and the proportion of women varied between 12% and 58%. Comorbidity reporting was inconsistent, though smoking was commonly documented, while diabetes and COPD were less frequently reported. Reported SSI rates ranged widely (1.0%–21.4%). Study characteristics are summarized in Table 1 and Supplementary Table S1.

**Table 1:** Clinical and demographic characteristics of the 11 included studies in the meta-analysis.

Author	Year	Sample Size	Age (Years)	Gender (Famle/male)	Smoking (Yes/No)	Infections (%)	Diabetes (Yes/No)	COPD (Yes/No)	Follow up (years)
Assakran (8)	2024	274	>18	55/219	54/220	4.4	30/197	3/224	NA
Bhardwaj (9)	2024	29,834	57.17 (13.36)	14,331/15,503	4,525/25,309	4.8	NA	NA	10
Cheema (10)	2021	166	52∼66	59/107	2/164	1	45/121	11/155	2
Christou (14)	2022	21,976	64.8 (15.4)	2,446/19,530	3,213/18,763	17.5	1,069/20,907	NA	10
Gala (15)	2025	1,413	55.89 (12.82)	818/595	688/725	21.35	232/1,182	67/1,346	5
Harriott (24)	2021	359	22−94	36/323	92/267	3.6	24/335	NA	5
Ortega-Deballon (25)	2023	32,633	59.6 (14.7)	17,953/14,680	NA	NA	NA	NA	2
Poruk (12)	2,017	362	22−88	208/154	67/295	9.94	73/289	25/337	NA
Romero (26)	2022	3,936	53.0 (14.9)	2,258/1,678	592/3,344	2.6	186/3,750	90/3,846	5
Stremitzer (27)	2010	476	59 (13)	221/255	146/313	6.51	55/419	NA	5
Winsnes (28)	2016	379	18–88	299/80	35/344	5.94	34/345	NA	10

Age is reported as mean (SD), median (IQR), or range where available. SSI, surgical site infection; DM, diabetes mellitus; COPD, chronic obstructive pulmonary disease; NA, not available.

### 3.3 Risk of bias assessment

All included studies were evaluated with the NOS, with scores ranging from 6 to 8, indicating moderate to high methodological quality. No study was excluded due to quality concerns, and the evidence was considered suitable for meta-analysis (Table 2).

**Table 2:** Quality assessment of included studies using the Newcastle-Ottawa scale (NOS).

Author (Year)	Selection (0–4)	Comparability (0–2)	Outcome/Exposure (0–3)	Total Score (0–9)	Quality Grade
Assakran (8)	4	1	2	7	High
Bhardwaj (9)	4	2	2	8	High
Cheema (10)	3	1	2	6	Moderate
Christou (14)	4	2	2	8	High
Gala (15)	4	1	2	7	High
Harriott (24)	3	1	2	6	Moderate
Ortega-Deballon (25)	4	2	2	8	High
Poruk (12)	3	1	2	6	Moderate
Romero-Velez (26)	4	1	2	7	High
Stremitzer (27)	3	1	2	6	Moderate
Winsnes (28)	4	2	2	8	High

### 3.4 Meta-analysis of risk factor

#### 3.4.1 Gender (female vs. Male)

**Figure 2:**
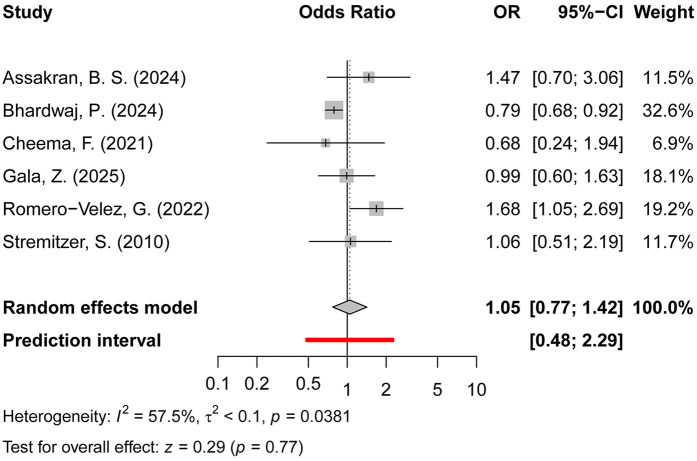
Forest plot of the association between gender (female vs. male) and SSI) following abdominal wall hernia repair.

Six studies (8–10, 15, 26, 27) investigated the impact of gender on postoperative outcomes. In the pooled analysis, female was associated with a significantly reduced risk of SSI and recurrence after abdominal wall hernia repair (OR = 1.05, 95% CI: 0.77–1.42, *P* = 0.77). The direction of effect was consistent across studies, with most individual estimates favoring lower SSI rates in females. The 95% PI ranged from 0.48 to 2.29, crossing the null value of 1.0, which indicates that the effect of gender on SSI risk varies across different clinical settings. Heterogeneity was low (*I*^2^ = 57.5%, *P* = 0.0381，tau^2^ < 0.10) (Figure 2), suggesting stability of the pooled result.

#### 3.4.2 Body mass index (obesity vs. non-obesity)

**Figure 3:**
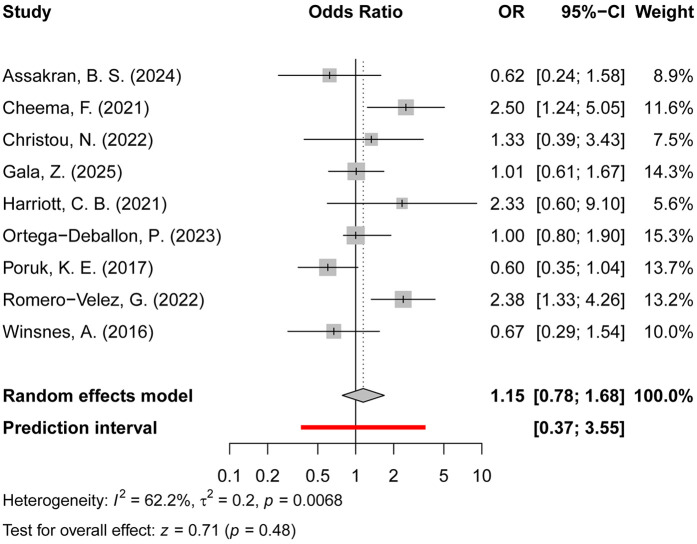
Forest plot of the association between obesity (BMI ≥ 30 kg/m^2^) and SSI after abdominal wall hernia repair.

A total of nine studies (8, 10, 12, 14, 15, 24–26, 28) evaluated the association between obesity and the risk of SSI and recurrence following abdominal wall hernia repair. The pooled odds ratio indicated that obesity was not significantly associated with SSI and recurrence (OR = 1.15, 95% CI: 0.78–1.68, *P* = 0.48). The 95% PI was 0.37–3.55, suggesting that the impact of BMI on infectious outcomes is inconsistent across the included cohorts. Statistical heterogeneity across studies was low to moderate (I^2^ = 62.2%, *P* = 0.0068, tau^2^ = 0.20) (Figure 3).

#### 3.4.3 Diabetes mellitus

**Figure 4:**
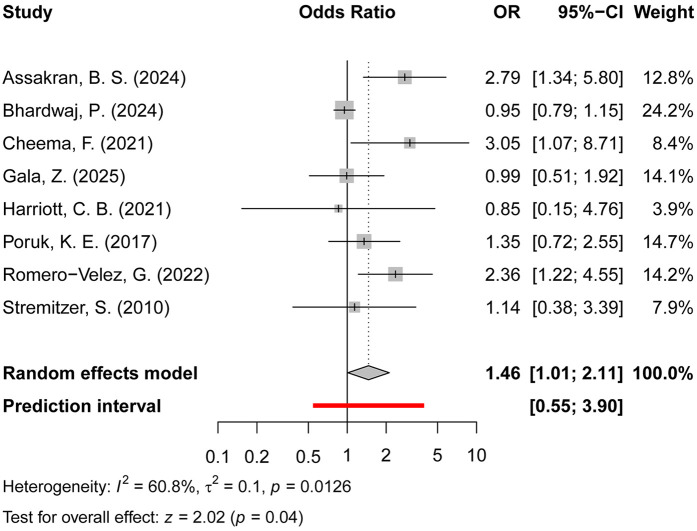
Forest plot of the association between diabetes mellitus and SSI following abdominal wall hernia repair.

Eight studies (8–10, 12, 15, 24, 26, 27) reported on the association between diabetes mellitus and the risk of SSI and recurrence. Meta-analysis showed no significant association between diabetes and SSI and recurrence after abdominal wall hernia repair (OR = 1.46, 95% CI: 1.01–2.11, *P* = 0.04). The 95% PI was 0.55–3.90, suggesting that the impact of Diabetes Mellitus on infectious outcomes is inconsistent across the included cohorts. Statistical heterogeneity was negligible (I^2^ = 60.8%, *P* = 0.0126, tau^2^ = 0.10) (Figure 4), indicating statistically significant associations across studies. Despite variability in study size and population, the effect estimates were largely clustered around the null value.

#### 3.4.4 COPD

**Figure 5:**
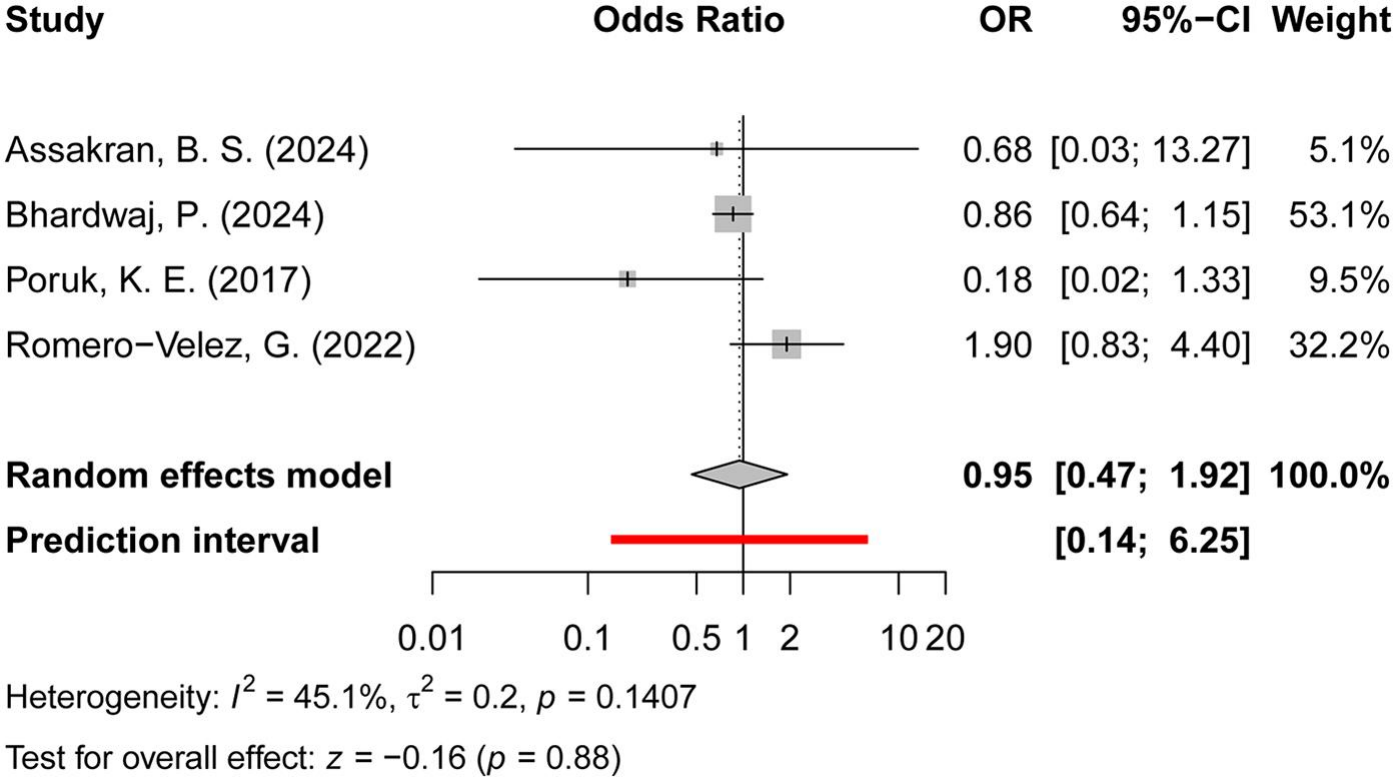
Forest plot of the association between COPD and SSI after abdominal wall hernia repair.

Four studies (8, 9, 12, 26) reported on the association between COPD and the risk of SSI and recurrence. The pooled odds ratio was 0.95 (95% CI: 0.47–1.92, *P* = 0.88), indicating no significant association. Heterogeneity across studies was low (I^2^ = 45.1%, *P* = 0.1407, tau^2^ = 0.2) (Figure 5). Furthermore, the calculated 95% PI ranged from 0.14 to 6.25. This exceptionally wide PI reflects substantial clinical variability and confirms that COPD is not a consistent, independent predictor for postoperative SSI in this patient population.

#### 3.4.5 Immunosuppressive therapy

**Figure 6:**
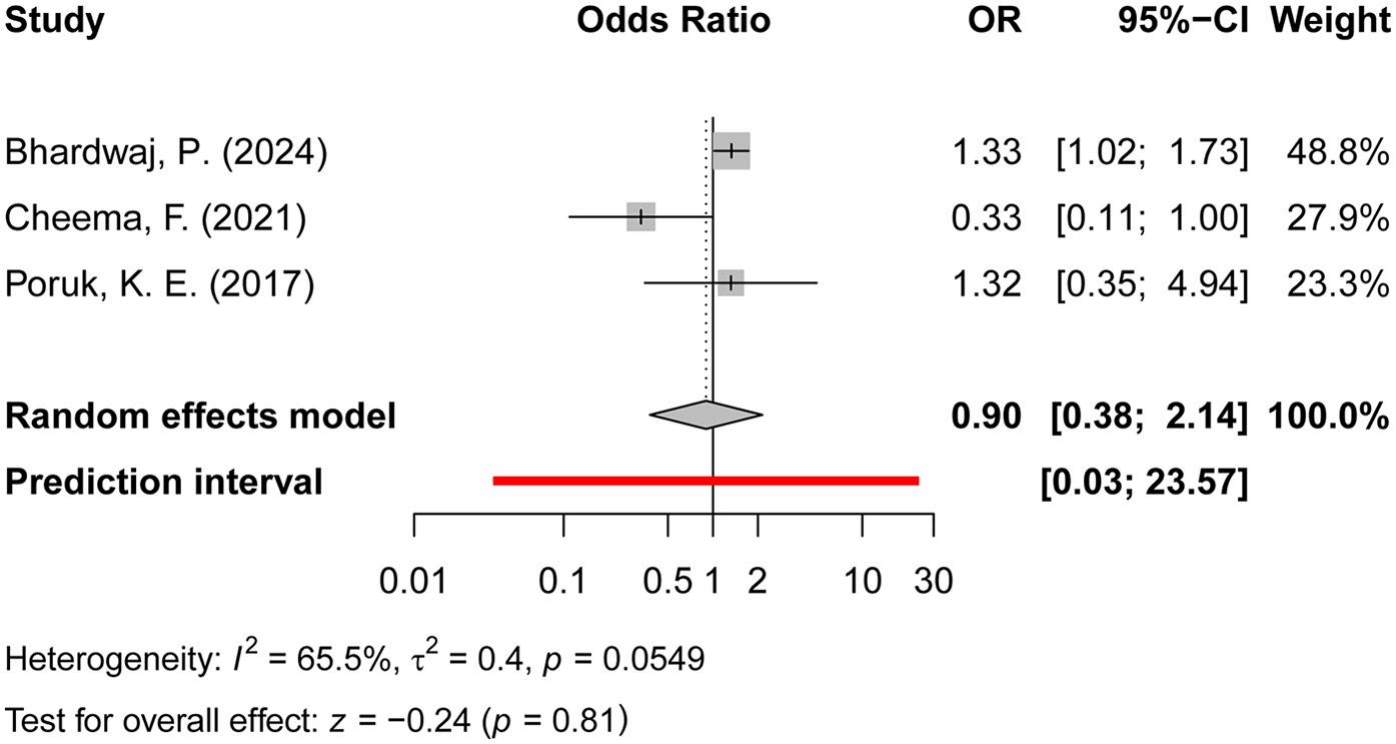
Forest plot of the association between immunosuppressive therapy and SSI after abdominal wall hernia repair.

Three studies (9, 10, 12) reported the association between immunosuppressive therapy and the risk of SSI and recurrence. The pooled odds ratio was 0.90 (95% CI: 0.38–2.14, *P* = 0.81), indicating no statistically significant association. However, there was substantial heterogeneity among studies (*I*^2^ = 65.5%, *P* = 0.0549, tau^2^ = 0.40) (Figure 6) suggesting variability in study populations or methodologies. Effect estimates varied considerably, with wide confidence intervals and inconsistent directions of effect.

#### 3.4.6 ASA Classification (>3 vs. <2).

**Figure 7:**
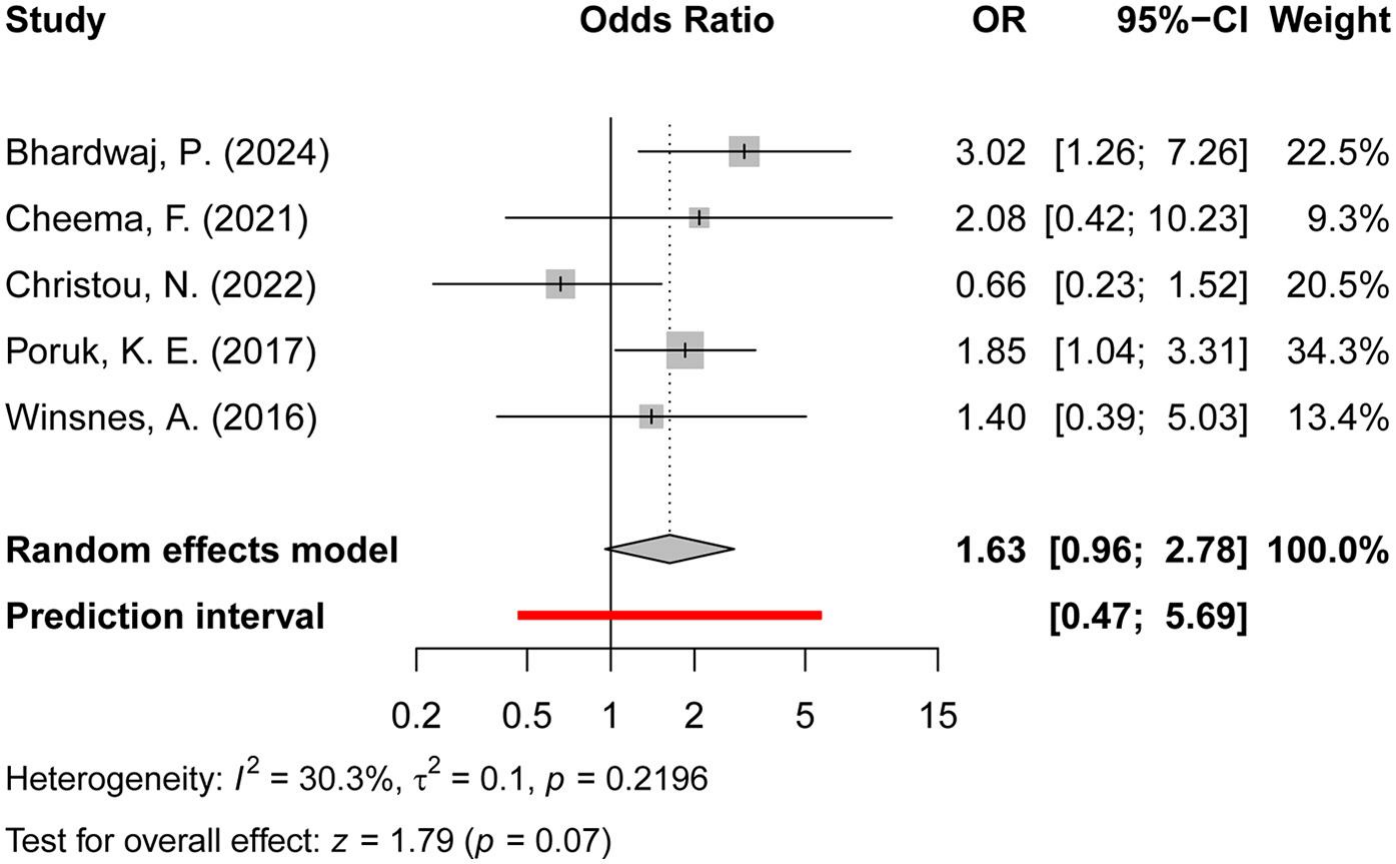
Forest plot of the association between ASA classification (>3 vs. <2) and SSI following abdominal wall hernia repair. The summary estimate (diamond) is calculated using a random-effects model (DerSimonian-Laird method). Each study is represented by a square, the size of which corresponds to its weight in the meta-analysis, and a horizontal line representing the 95% CI). The vertical solid line represents the null effect (OR = 1.0). The I^2^ value and *P*-value for the Cochrane Q test indicate the level of statistical heterogeneity. OR, odds ratio; CI, confidence interval.

Five studies (9, 10, 12, 14, 28) reported on the association between ASA classification and the risk of SSI and recurrence. Meta-analysis showed that a high ASA class (>3) was not significantly associated with SSI and recurrence after hernia repair (OR = 1.63, 95% CI: 0.96–2.78, *P* = 0.07). Moderate heterogeneity was observed across studies (*I*^2^ = 30.3%, *P* = 0.2196, tau^2^ = 0.10) (Figure 7). The direction of effect varied, and confidence intervals were wide in some studies, limiting the interpretability of the pooled estimate. The calculated 95% PI ranged from 0.47 to 5.69 suggests that while higher ASA scores are often clinical indicators of frailty, they do not consistently predict SSI risk across all clinical scenarios within the analyzed evidence framework.

#### 3.4.7 Hernia recurrence

Quantitative data specifically for hernia recurrence was notably scarce across the included studies. Only one study (10) provided granular multivariate analysis for a “2-year composite recurrence” endpoint. In their cohort of 500 patients, several risk factors showed specific associations with recurrence: obesity (BMI ≥ 30) was associated with an adjusted Odds Ratio (aOR) of 1.48 (95% CI: 0.94–2.34), while Diabetes Mellitus (DM) showed an aOR of 1.41 (95% CI: 0.81–2.46). Notably, preoperative hypoalbuminemia (<3.5 g/dL) was a significant predictor for recurrence (aOR = 2.45, 95% CI: 1.15–5.22). However, because these specific, factor-level odds ratios for recurrence were absent in the remaining 10 studies (which either did not report recurrence or reported it only as a descriptive total), a formal meta-analysis could not be performed. The lack of comparable numerical data across the literature precludes the generation of a pooled estimate for these variables.

### 3.5 Sensitivity analysis

To assess the robustness of the meta-analysis results, sensitivity analyses were performed by sequentially omitting one study at a time (leave-one-out method). The overall effect sizes for all primary outcomes (SSI and recurrence) remained stable, indicating that no single study had a disproportionate influence on the pooled estimates. Additional analyses using fixed-effect and random-effect models yielded consistent conclusions.

### 3.6 Publication bias

Publication bias was assessed using Egger's regression test for the primary outcomes. For SSI and recurrence -related analyses, no significant publication bias was detected (Egger's test: *P* = 0.274). Similarly, for hernia recurrence, Egger's test indicated no evidence of bias (*P* = 0.311). Due to the small number of studies included for several risk factors (e.g., COPD, immunosuppressive therapy, ASA classification), publication bias could not be reliably assessed in those subgroups.

## 4 Discussion

This systematic review and meta-analysis comprehensively evaluated patient-related risk factors associated with hernia recurrence and SSI following abdominal wall hernia repair. Across the 11 included studies, we analyzed key host variables including gender, BMI, diabetes mellitus, COPD, immunosuppressive therapy, and ASA classification. The results revealed that female was significantly associated with lower SSI risk, whereas other factors showed variable or statistically non-significant associations with adverse outcomes.

Regarding gender-related outcomes, our pooled random-effects analysis showed that female sex was not significantly associated with lower SSI risk. This finding contrasts with several earlier retrospective cohort studies, including some ACS-NSQIP analyses, which reported a modestly lower SSI rate among female patients (8, 9). Although several biological explanations have been proposed, such as lower visceral fat accumulation, differences in skin microbiota, and hormone-mediated immune modulation favoring wound healing in females (10, 14, 15, 24)，our results are more consistent with recent large-scale studies showing no significant sex-based differences after adjustment for confounding factors. Importantly, the wide 95% prediction interval (0.48–2.29) indicates substantial between-study variability, suggesting that the effect of sex may differ across surgical populations and clinical settings and is therefore unlikely to serve as a reliable independent predictor of postoperative complications. Similarly, obesity was not significantly associated with SSI in our pooled analysis. This finding differs from conventional surgical assumptions and from several earlier multicenter studies that identified BMI > 30 kg/m^2^ as a risk factor for wound morbidity (9, 10, 29). Several factors may explain this discrepancy in contemporary practice, including standardized perioperative glucose control, more appropriate weight-based prophylactic antibiotic dosing, and the growing use of minimally invasive techniques, such as laparoscopic and robotic surgery, which may reduce SSI risk in obese patients by avoiding large incisions. In addition, the effect of obesity may depend on operative approach, with excess risk more commonly observed in open procedures. However, the limited granularity of the primary studies precluded stratified analysis by surgical technique.

Diabetes mellitus, another commonly reported SSI risk factor, also did not reach statistical significance in our meta-analysis (24). This finding aligns with recent large-scale studies, which suggest that well-controlled diabetic patients may not carry excess risk if appropriate antibiotic prophylaxis and glycemic control are ensured (9). Notably, prior studies often failed to distinguish between controlled and uncontrolled diabetes or type 1 vs. type 2, which may partially explain conflicting results in literature. Similarly, COPD was not significantly associated with SSI in our analysis, despite theoretical rationale linking chronic coughing, hypoxia, and poor wound healing to higher infection risk (9, 10, 12). This differs from the findings, which reported higher SSI rates in COPD patients following abdominal surgery. The heterogeneity in our analysis was substantial, reflecting variation in diagnostic criteria for COPD and differences in surgical techniques, particularly the extent of muscle dissection and use of drains. Future studies should stratify COPD by severity and treatment status. Regarding immunosuppression, while the pooled effect estimate suggested a slightly increased SSI risk, it was not statistically significant. This contrasts with guidelines from the European Hernia Society (EHS), which consider immunocompromised patients at increased risk of both SSI and recurrence (30). However, many studies failed to report the type, duration, and intensity of immunosuppressive therapy, making it difficult to draw strong conclusions. In addition, immunosuppressed patients may have been excluded from elective hernia repair in some cohorts, introducing selection bias. High ASA class (>3) is typically used as a surrogate for frailty and comorbidity burden. Surprisingly, our meta-analysis did not identify a significant association between ASA classification and SSI (8, 9, 12, 26). This differs from previous work, it identified ASA >2 as an independent predictor of SSI and reoperation. One explanation may be that ASA is a crude marker, and more granular comorbidity indices such as the Charlson Comorbidity Index (CCI) may offer better discrimination.

Our study has several strengths. First, it is the first to systematically evaluate multiple patient-related risk factors for both hernia recurrence and SSI using rigorous methodology. Second, our search strategy covered both Western and Chinese databases, improving inclusiveness and external validity. Third, quality assessment using the NOS showed that most included studies were of moderate to high quality. However, some limitations should be acknowledged. First, most of the included studies were observational in nature, which introduces a risk of residual confounding despite adjustments in the original analyses. Additionally, outcome definitions—particularly for hernia recurrence—were not standardized across studies. Second, key procedural variables such as mesh type, fixation technique, contamination class, surgical approach (e.g., open vs. laparoscopic), and use of surgical drains were inconsistently reported and could not be quantitatively analyzed. This limits our ability to evaluate the influence of these technical factors on SSI or recurrence. Third, due to the limited number of studies for several risk factors, we were unable to formally assess publication bias using funnel plots or statistical tests, which may affect the consistent of the findings. Lastly, some studies reported SSI and hernia recurrence as a combined outcome, rather than distinguishing them. This precluded separate analyses of infectious vs. mechanical complications and limited our ability to determine whether specific risk factors (e.g., diabetes mellitus or obesity) are more associated with one outcome over the other.

In conclusion, our analysis suggests that female may be associated with lower SSI risk after abdominal wall hernia repair, while other common host-related factors such as obesity, diabetes, COPD, and high ASA class do not consistently predict adverse outcomes. These findings underscore the need for individualized preoperative risk stratification rather than reliance on isolated demographic or comorbidity factors. Future high-quality prospective studies are needed to integrate both patient and surgical variables into robust predictive models.

## Data Availability

The raw data supporting the conclusions of this article will be made available by the authors, without undue reservation.
