# Supplementary material for: Risk factors for recurrence and surgical site infection after abdominal wall hernia repair: a systematic review and meta analysis
Source: Front Surg. 2026 Jun 3;13:1835070. doi: 10.3389/fsurg.2026.1835070 (PMC13272115; doi:10.3389/fsurg.2026.1835070)
Supplement: Supplementary file 1 [file Table1.docx]

| **Author** | **Year** | **Gender (F) OR (95% CI)** | **Obesity OR (95% CI)** | **DM OR (95% CI)** | **Immunosuppressants OR (95% CI)** | **COPD OR (95% CI)** | **ASA >3 OR (95% CI)** |
| --- | --- | --- | --- | --- | --- | --- | --- |
| **Assakran, B. S.** | 2024 | 1.47 (0.70–3.06) | 0.62 (0.24–1.58) | 2.79 (1.34–5.80) | – | 0.68 (0.03–13.27) | – |
| **Bhardwaj, P.** | 2024 | 0.79 (0.68–0.92) | – | 0.95 (0.79–1.15) | 1.33 (1.02–1.73) | 0.86 (0.64–1.15) | 3.02 (1.26–7.26) |
| **Cheema, F.** | 2021 | 0.68 (0.24–1.94) | 2.50 (1.24–5.05) | 3.05 (1.07–8.71) | 0.33 (0.11–1.00) | – | 2.08 (0.42–10.23) |
| **Gala, Z.** | 2025 | 0.99 (0.60–1.63) | 1.01 (0.61–1.67) | 0.99 (0.51–1.92) | – | – | – |
| **Romero-Velez, G.** | 2022 | 1.68 (1.05–2.69) | 2.38 (1.33–4.26) | 2.36 (1.22–4.55) | – | 1.90 (0.83–4.40) | – |
| **Stremitzer, S.** | 2010 | 1.06 (0.51–2.19) | – | 1.14 (0.38–3.39) | – | – | – |
| **Christou, N.** | 2022 | – | 1.33 (0.39–3.43) | – | – | – | 0.66 (0.23–1.52) |
| **Harriott, C. B.** | 2021 | – | 2.33 (0.60–9.10) | 0.86 (0.15–4.76) | – | – | – |
| **Ortega-Deballon, P.** | 2023 | – | 1.00 (0.80–1.90) | – | – | – | – |
| **Poruk, K. E.** | 2017 | – | 0.60 (0.35–1.04) | 1.35 (0.72–2.55) | 1.32 (0.35–4.94) | 0.18 (0.02–1.33) | 1.85 (1.04–3.31) |
| **Winsnes, A.** | 2016 | – | 0.67 (0.29–1.54) | – | – | – | 1.40 (0.39–5.03) |

**Table S1. Odds Ratios (ORs) for Risk Factors Associated with SSI and/or Hernia Recurrence, Stratified by Study**

Note: OR = Odds Ratio; CI = Confidence Interval; DM = Diabetes Mellitus; COPD = Chronic Obstructive Pulmonary Disease; ASA = American Society of Anesthesiologists physical status classification. “–” indicates data not reported in the respective study.
